# Role of Planetary Health Diet in the association between genetic susceptibility to obesity and anthropometric measures in adults

**DOI:** 10.1038/s41366-024-01656-7

**Published:** 2024-10-17

**Authors:** Tiina Suikki, Mirkka Maukonen, Heidi Marjonen-Lindblad, Niina Erika Kaartinen, Tommi Härkänen, Pekka Jousilahti, Anne-Maria Pajari, Satu Männistö

**Affiliations:** 1 Finnish Institute for Health and Welfare, P.O. Box 30, 00271 Helsinki, Finland; 2 University of Helsinki, Helsinki, Finland

**Keywords:** Risk factors, Obesity, Epidemiology

## Abstract

**Background/Objective:**

The roles of overall diet quality in linking genetic background with anthropometric measures are unclear, particularly regarding the recently developed Planetary Health Diet (PHD). This study aims to determine if the PHD mediates or moderates the relationship between genetic susceptibility to obesity and anthropometric measures.

**Subjects/Methods:**

The study involved 2942 individuals from a Finnish population-based cohort (54% women, mean age 53 (SD ± 13) years). Habitual diet was assessed using a validated 130-item food frequency questionnaire, and the PHD Score (total score range 0–13 points) was adapted for Finnish food culture to evaluate diet quality. Genetic susceptibility to obesity was evaluated with a polygenic risk score (PRS) based on one million single nucleotide polymorphisms associated with body mass index (BMI). Baseline anthropometrics included weight, height, waist circumference (WC), and body fat percentage, with changes in these measures tracked over 7 years. A five-step multiple linear regression model and multivariable logistic regression with interaction terms were used to assess the mediating and moderating effects of the PHD. These analyses were also replicated in another Finnish cohort study (2 834 participants).

**Results:**

PRS for BMI was positively associated with baseline BMI and changes in anthropometric measures, except waist circumference (*p* = 0.12). Significant associations were observed for baseline BMI and WC (*p* < 0.001), changes in BMI and WC (*p* = 0.01), and body fat percentage change (*p* = 0.05). However, the PHD (average score 3.8 points) did not mediate or moderate these relationships. These findings were consistent in the replication cohort.

**Conclusion:**

Diet quality assessed with the PHD did not mediate or moderate the associations between genetic susceptibility to obesity and anthropometric measures. This lack of effect may be partly due to low adherence to the PHD and the older age of participants ( > 50 years) at baseline.

## Introduction

Obesity is a complex condition characterised by excessive fat accumulation, resulting from the interplay between genetic and lifestyle factors such as nutrition, physical activity, and sleep [1]. It is a significant risk factor for numerous comorbidities, including type 2 diabetes, cardiovascular diseases, and various cancers [2].

The heritability of obesity has been estimated as approximately 40−75% [3, 4]. While rare monogenic traits contribute to weight changes and obesity (monogenic obesity), the most common form of obesity arises from multiple independent gene variants known as single nucleotide polymorphisms (SNP) across the genome (polygenic obesity) [5]. Based on these SNP, a person’s susceptibility to obesity can be analysed with genetic risk scores, in which the effect of one independent SNP is small, but as the number of variants rises, the risk of obesity increases [5]. Even though the number of known SNP associated with obesity, especially body mass index (BMI), has increased to over two million variants due to new and more accurate analysing methods, they explain <10% of the variation in BMI between individuals [5, 6]. This suggests that individuals with a high genetic risk for obesity do not gain more weight compared to those with a lower risk for obesity and that other factors including environment and food choices and food availability influence the variation between genetic risk scores and anthropometric measures.

Different approaches can be used to examine gene-environment interactions in obesity. While mediation analyses provide information on the mechanisms of how genes may influence anthropometric measures, effect modification (moderation or interaction) analyses provide information on the subjects affected by the moderators, such as overall diet quality [7]. In previous research, eating behaviours, such as snacking, emotional eating, and uncontrolled eating have appeared to mediate the association between genes and anthropometric measures [8–10]. Eating behaviours are also closely linked to overall diet quality. As overall diet quality considers the synergic interactions between single food groups and nutrients, research on it may enable a more comprehensive understanding of the relationship between diet and health [11]. Furthermore, for example, high consumption of fruits and vegetables, and high intake of fibre have been found to attenuate the genetic risk of obesity while high consumption of sugar-sweetened beverages and fried foods seem to pronounce it [12–15]. However, only few studies have, so far, examined the mediating and modifying roles of overall diet quality on the associations between genetic susceptibility to obesity and anthropometric measures, and the results have been inconsistent [9, 15–21].

In 2019, the EAT-*Lancet* Commission published the Planetary Health Diet (PHD), which is a global reference diet promoting both human health and environmental sustainability within planetary boundaries [22]. The PHD also embeds the aim of decreasing global obesity rates [22]. So far, the findings regarding associations between the PHD and obesity measures have been conflicting [23–29]. Furthermore, the role of the PHD on the association between genetic susceptibility to obesity and anthropometric measures has not been studied [22].

Therefore, we examined retrospectively whether the PHD mediates or moderates the associations between genetic susceptibility to obesity and anthropometric measures (weight, BMI, waist circumference (WC), and percentage of body fat (body fat %)) in the Finnish general adult population. We hypothesised that better adherence to the PHD could offset the genetic susceptibility to obesity in a 7-year follow-up.

## Methods

### Data source

We used data from the Finnish population-based comprehensive health examination study, DIetary Lifestyle and Genetic Determinant of Obesity and Metabolic Syndrome (DILGOM) 2007 Study, and its 7-year follow-up, DILGOM 2014 [30, 31]. DILGOM 2007 comprised 5 024 subjects (participation rate 80%). In 2014, all eligible participants in DILGOM 2007 (*n* = 4581) were invited to DILGOM 2014 [30], and of these 3735 participated (participation rate 82%) in two groups. The participants of the group 1 (*n* = 1 312) attended the clinical examination, whereas participants in the group 2 (*n* = 2423) received a measuring tape for anthropometric self-assessments via mail to complete at home [31].

We replicated analyses in another Finnish population-based study, the Health 2000 Study (Health 2000) and its 11-year follow-up Health 2011 [32, 33]. Health 2000 comprised 6 771 subjects who participated in health examinations and interviews (participation rate 84%) [32]. All participants in Health 2000 were invited for the follow-up phase in 2011 (Health 2011). Of the invited, 4006 subjects (63%) attended [33].

In all studies, data was gathered with self-administered questionnaires including food frequency questionnaire (FFQ), interviews, clinical examinations or self-measurements, and blood samples. All studies were carried out by the Finnish Institute for Health and Welfare and conducted according to the guidelines of the Declaration of Helsinki. The research protocols were approved by the Ethics Committee of the Hospital District of Helsinki and Uusimaa (DILGOM 2007: 229/EO/2006, DILGOM 2014: 332/13/03/00/13, Health 2000: 407/E3/2000, Health 2011: 45/13/03/00/11). All participants gave written informed consent.

### Study population

We included subjects with completed FFQ at baseline, body weight and height at baseline and follow-up, and PRS_BMI_. To control for implausible total energy intake, we excluded participants within 0.5% of either end of the total energy intake distribution (DILGOM 2007; *n* = 48) [34], or participants with total energy intake values < 600 and > 7000 kcal (Health 2000; *n* = 18). In addition, all pregnant women (DILGOM 2007/2014, *n* = 7; Health 2000/2011, *n* = 9) were excluded. The final study population comprised 2942 participants from DILGOM 2007 and 2014, and 2834 participants from Health 2000 and 2011.

### Dietary intake and diet quality

Participants’ habitual diet was assessed using an FFQ which is repeatedly updated and validated against food records in Finnish adult populations [35, 36]. Participants completed the FFQ either at the study site or at home and returned it to the Finnish Institute for Health and Welfare via mail. The FFQ recorded retrospective dietary intake for the previous 12 months and including 128−130 food items (depending on datasets) representing commonly consumed foods and beverages in Finland, identified from the most recent food consumption surveys (e.g., FinDiet). These items were linked to the national food composition database (Fineli^®^). The frequency of consumption was assessed with nine categories ranging from “never or seldom” to “six or more times a day” with predefined portion sizes (e.g., slice or glass, serving). The in-house software FINESSI then aggregates this data to create a final dataset of average daily food consumption, nutrient intake, and energy intake comprising approximately 80 ingredient groups, 80 food groups, and 100 nutrients [37].

The average daily dietary intake data gathered from FFQ was used to compose 13 food group and nutrient components included in the dietary index based on the PHD (Planetary Health Diet Score, PHDS) adapted for Finnish food culture [29]. These components included whole grains, all vegetables, potatoes, fruits and berries, dairy foods, red and processed meat, chicken, eggs, fish, legumes, nuts and seeds, ratio of unsaturated and saturated fat intake, and sucrose. Each participant’s mean daily consumption of these components (except for fat ratio) was energy-standardised by dividing the consumption in grams by the participant’s mean daily total energy intake (kcal) and multiplying by 2500, the recommended total energy intake in the original PHD [22]. The energy-standardised consumption values were then compared to the target values set in the PHDS to assess overall diet quality considering simultaneously the human health and environmental sustainability. Each component was assigned one point if its consumption met the target value, or zero points if it did not meet the target value [29]. Thus, the total score could range from 0 to 13 points, with a higher total score indicating better adherence to the PHD.

### Anthropometric variables

Anthropometric variables (weight, height, WC, and body fat%) were measured either by trained study nurses at the study site or self-reported (weight and height) and self-measured (WC) by the participant (group 2 in DILGOM 2014 Study) according to the standardised protocols, wearing light clothes and no shoes [38]. Precise measuring protocols are described elsewhere [31–33, 39]. The validity between the self-reported or self-measured anthropometrics and the measures taken by the nurses has been approved previously [31]. The body fat% was utilised only from DILGOM studies, in which the same electric bioimpedance scale (TANITA TBF-300MA; Tanita Corporation of America, Inc) was applied at baseline and follow-up [31].

Changes in anthropometric measures were calculated as an absolute change by subtracting baseline measurement from follow-up measurement, or as a percentual change by dividing absolute anthropometric change by baseline measurement, multiplied by 100. Percentual weight changes were analysed as a continuous or dichotomous variable ( < 5% or ≥ 5% change in weight, BMI, WC, or body fat%).

### PRS for BMI

The DNA from the DILGOM 2007 and Health 2000 participants was isolated from whole blood samples, which were analysed as part of the laboratory examination at the Department of Molecular Medicine in Kansanterveyslaitos (nowadays Finnish Institute for Health and Welfare). Further, the samples were genotyped using various Illumina arrays (HumanCoreExome, Human610, HumanOmniExpress: Illumina Inc., San Diego, and Thermo Fisher Scientific, Santa Clara, CA, USA), and genotype calls were made with the GenCall or zCall algorithm. Genotype imputation was carried out at the Institute for Molecular Medicine Finland (FIMM) as part of the FinnGen project [40]. The haplotypes were estimated using Eagle 2.3.5 [41], and imputation was performed with Beagle 4.1 [42] using a high-coverage, population-specific SISu v3 reference panel of 3775 whole-genome sequences.

The PRS_BMI_ to represent genetic susceptibility to obesity was derived from a previous UK Biobank genome-wide association study (*n* = 439,590) [43]. The PRS was calculated with the continuous shrinkage (PRS-CS) method, which utilises a Bayesian regression framework and considers the weighted involvement of different SNP across the genome to the variation of BMI [44]. The European 1000 Genomes Project samples were used as an external LD reference panel [44, 45]. PLINK’s –score command was used to complete the PRS for each participant [45]. The mean of the PRS was scaled to zero with R 3.6.0 to reflect SD scaling in addition to mean centreing. In the current study, the PRS_BMI_ included 959 569 SNP.

### Other factors

Covariates (age, sex, educational level, leisure-time physical activity, and smoking status), and factors used as exclusion criteria in sensitivity analyses (back pain, arthritis, cancer, diabetes), and depression or any other mental illness commonly known to associate with weight changes [46, 47], were obtained from self-administered questionnaires.

To assess educational level, in DILGOM 2007 participants were asked to report the total number of school years, which were then categorised into tertiles (low, middle, high) according to sex and birth cohort to adjust for the extension of the basic education system and increase in average school years over the last decades. In Health 2000, participants reported their educational level according to the following categories: lower than upper secondary school or vocational school (low), graduated from upper secondary school or vocational school (intermediate) or graduated from university or university of applied sciences (high). Leisure-time physical activity was assessed by giving four options: inactive (light activities, like reading and watching television), moderately active (e.g., walking, cycling, and gardening at least 4 h/wk), active (e.g., brisk running, swimming, or other physically demanding activities at least 3 h/wk) or very active (competitive sports or other physically demanding exercise several times/wk). In the final data, categories ”active” and ”very active” were combined due to the low number of participants in the latter category. Smoking status was asked using a four-level classification: never a smoker, quit > ½ year ago, quit < ½ year ago or current smoker in the questionnaires. The categories “quit > ½ year ago” and “quit < ½ year ago” were combined to the one category “former smoker” during data processing.

### Statistical methods

All statistical analyses were performed with SPSS statistical computing software version 27.0 (IBM SPSS Statistics, IBM Corp.). A p value of < 0.05 was considered statistically significant. Men and women were analysed together since, in general, no significant interactions emerged between PRS_BMI_ and sex on changes in anthropometrics. Variables that did not satisfy the normality assumption were log-transformed with the natural logarithm. We present the baseline and follow-up descriptive statistics from all participants and according to PRS_BMI_ quintiles as means with standard deviations for continuous variables or as percentages for categorical variables.

In the mediation analysis, we used five-step multiple linear regression models according to the Baron and Kenny mediation test to determine whether the baseline PHDS mediates the association between PRS_BMI_ and anthropometric measures (baseline BMI and WC, and changes in weight, BMI, WC, and body fat%) [48]. In step 1, the linear association between PRS_BMI_ (independent variable) and anthropometrics (dependent variables) was tested; in step 2, the association between PRS_BMI_, and PHDS (mediator) was tested; in step 3, the associations between the mediator and the dependent variables were analysed; in step 4, the linear regression in step 3 was controlled with the independent variable (PRS_BMI_); and in step 5, the association in step 1 was controlled with the mediator (PHDS). After controlling with the PHDS, we expected the association in step 1 to be attenuated as the PHDS was presumed to explain the underlying mechanism linking genetic susceptibility for obesity to anthropometric measures. However, before the mediation analyses are performed, linear relationships between the main variables (PRS_BMI_, anthropometric variables, and PHDS) need to be fulfilled.

In the moderation (interaction) analyses, we determined whether the baseline PHDS modified the associations between the PRS_BMI_ and each anthropometric measures by including an interaction term (PRS_BMI_ in quintiles * PHDS in tertiles) into the main model for each outcome (baseline BMI and WC, and anthropometric changes in weight, BMI, WC and body fat%). Interaction studies enable the identification of subgroups that may benefit more or be more sensitive to exposure factors such as diet quality [7]. We used multivariate logistic regression to examine associations between the PRS_BMI_ and baseline anthropometric measures, and anthropometric changes when participants were stratified according to the PHDS tertiles.

Statistical analyses were carried out with two main models. In the first model (model 1), the associations were adjusted for baseline age, sex, and baseline anthropometric measures depending on which anthropometric change variable (weight, BMI, WC, or body fat%) was examined. In addition, analyses on weight change were adjusted for baseline height. In model 2, model 1 was further adjusted for baseline educational level, leisure-time physical activity, and smoking status.

Furthermore, we carried out two sensitivity analyses. In the first analysis, we excluded participants who reported at baseline or follow-up any illness that might influence weight (model 2). In the second analysis, we excluded energy under-reporters (DILGOM 2007, *n* = 616; Health 2000, *n* = 838; model 2). Baseline energy under-reporting was defined based on the ratio of reported energy intake and predicted basal metabolic rate, where the ratio ≤ 1.14 indicated energy under-reporting [49, 50].

## Results

### Selected characteristics of participants

**Fig. 1:**
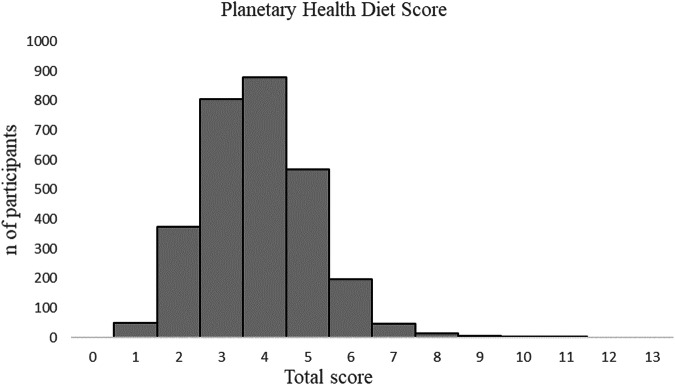
Baseline distribution of Planetary Health Diet Score in DILGOM 2007 data (*n* = 2942). Total score ranged from 0 to 11 points when theoretical maximum could have been 13 points (mean 3.8 points, SD 1.3).

In total, the DILGOM 2007 and 2014 data sample included 2942 participants (54% of women) with a mean age of 53 years (SD ± 13 years) at baseline (Table 1). In every PRS_BMI_ quintile, the mean baseline age was similar. Participants with the lowest susceptibility for obesity (the first PRS_BMI_ quintile) were less often in the lowest educational level and current smokers compared to the other quintiles. The PHDS ranged from 1 to 11 points among all participants (Fig. 1), but the mean PHDS and mean daily energy intake did not differ across the PRS_BMI_ quintiles (Table 1).

**Table 1:** Baseline and follow-up characteristics of all participants and participants according to polygenic risk score for BMI (PRS_BMI_) quintiles in DILGOM 2007 and 2014 Studies.

	All	PRS_BMI_				
		1	2	3	4	5
Number of participants (%)	2942	590 (20)	600 (20)	594 (20)	602 (21)	556 (19)
Women (%)	1595 (54)	298 (51)	309 (52)	342 (58)	331 (55)	315 (57)
**Baseline**						
Age, years	53 (13)	53 (13)	53 (13)	53 (13)	53 (13)	52 (13)
Low education level^a^, %	27	22	29	26	29	30
Current smokers, %	16	12	14	17	19	17
Leisure-time physical inactivity^b^, %	17	16	17	17	15	20
Energy intake, kJ	10,457 (3761)	10,463 (3746)	10,610 (3773)	10,592 (3745)	10,323 (3806)	10,286 (3735)
Planetary Health Diet Score^c^	3.8 (1.3)	3.8 (1.3)	3.8 (1.3)	3.9 (1.3)	3.8 (1.3)	3.9 (1.3)
1st tertile (1–3 points), %	42	44	42	40	44	40
2nd tertile, (4 points), %	30	30	33	27	30	31
3rd tertile, (5–11 points), %	28	27	26	33	26	30
Height, cm	168.7 (9.1)	169.8 (9.0)	169.4 (9.2)	168.4 (9.3)	168.2 (9.0)	167.6 (8.9)
Weight, kg	76.3 (15.2)	72.4 (13.2)	74.4 (14.2)	75.6 (13.8)	77.4 (15.1)	82.0 (17.6)
BMI, kg/m^2^	26.8 (4.7)	25.0 (3.7)	25.9 (4.1)	26.6 (4.2)	27.3 (4.6)	29.1 (5.7)
Waist circumference, cm						
Men	96.2 (11.5)	92.3 (10.4)	94.5 (10.9)	95.8 (10.5)	97.8 (11.4)	101.8 (12.5)
Women	86.2 (13.1)	81.8 (10.4)	84.4 (11.8)	85.6 (11.9)	86.9 (12.9)	91.8 (15.7)
Body fat, %						
Men	24.7 (6.6)	22.6 (6.1)	23.6 (6.1)	24.5 (6.1)	25.5 (6.4)	27.8 (7.2)
Women	35.1 (7.4)	33.0 (6.7)	34.0 (7.2)	35.2 (7.3)	35.6 (7.2)	37.8 (7.7)
**Follow-up**						
Weight change, %	0.8 (5.9)	2.2 (5.2)	0.9 (5.4)	0.9 (5.6)	0.9 (6.1)	0.6 (7.2)
*n* ≥ 5% (%)	794 (27)	155 (26)	155 (26)	172 (29)	163 (27)	149 (27)
BMI change, kg/m^2^	0.1 (2.1)	0.1 (1.8)	0.1 (2.0)	0.2 (2.0)	0.2 (2.2)	0.0 (2.6)
*n* ≥ 5% (%)	704 (24)	138 (23)	131 (22)	155 (26)	147 (24)	133 (24)
Waist circumference change, cm^d^	1.8 (5.9)	1.7 (5.3)	1.7 (5.2)	1.9 (6.1)	1.7 (6.1)	1.7 (6.3)
*n* ≥ 5% (%)	334 (31)	74 (31)	69 (29)	70 (34)	63 (30)	58 (30)
Body fat change, %-point^e^	1.0 (3.6)	1.0 (3.5)	0.8 (3.1)	1.1 (3.9)	1.0 (3.7)	1.1 (3.2)
*n* ≥ 5% (%)	444 (44)	105 (45)	97 (42)	88 (46)	79 (41)	75 (43)

*kJ* kiloJoules, *BMI* body mass index.

Data is presented as means with standard deviations (SD) for continuous variables or percentages for categorical variables.

^a^Lowest tertile of self-reported total school years according to birth cohort to adjust for the extension of the basic education system and the increase of average school years over time.

^b^Leisure-time physical inactivity; light activities, like reading and watching television.

^c^Score could range from 0 to 13 points.

^d^*n* = 1081, PRS quintiles: n_1_ = 236, n_2_ = 239, n_3_ = 208, n_4_ = 207, n_5_ = 191.

^e^*n* = 1020, PRS quintiles: n_1_ = 231, n_2_ = 230, n_3_ = 192, n_4_ = 192, n_5_ = 175.

All baseline anthropometrics (weight, BMI, WC, and body fat%) increased towards the highest PRS_BMI_ quintile (genetic susceptibility to obesity increased) (Table 1). For example, the mean BMI was 4.1 units higher in the highest PRS_BMI_ quintile compared to the lowest quintile, and the mean WC was almost 10 cm wider in the fifth quintile compared to the first quintile both in men and women. However, changes in anthropometrics during the 7-year follow-up were not substantially different across the quintiles of PRS_BMI_ (Table 1).

### Mediation analysis

**Fig. 2:**
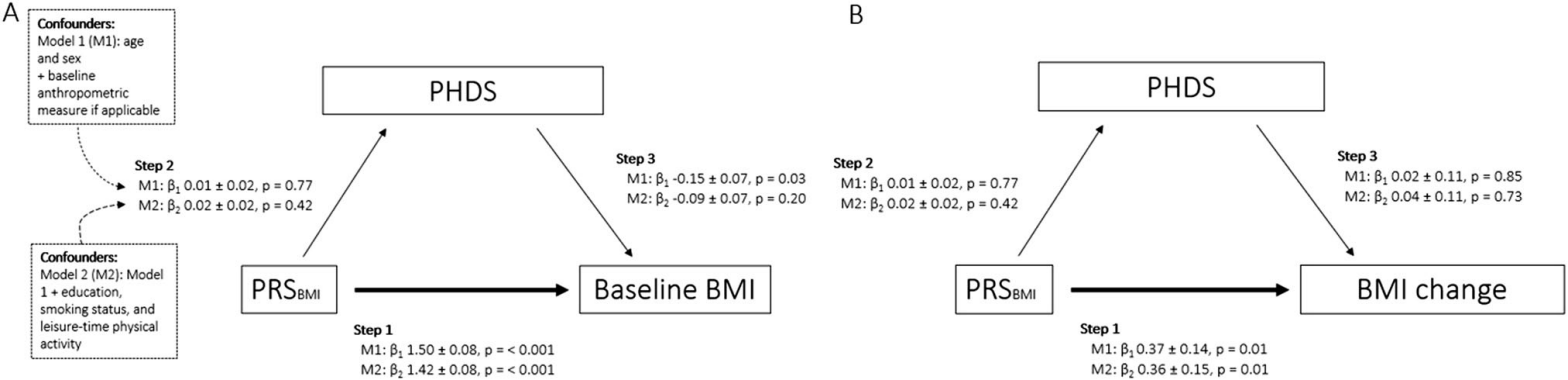
Linear associations between polygenic risk score for BMI (PRS_BMI_), Planetary Health Diet Score (PHDS), and anthropometric measures in DILGOM 2007 and 2014 Studies. Unstandardised coefficients (β1, Model 1, and β2, Model 2) for linear regression analyses between main variables; polygenic risk score for body mass index, PRS_BMI_, Planetary Health Diet Score (PHDS) and anthropometric measures; **A** baseline body mass index (BMI) and **B** percentual anthropometric change measured with BMI in DILGOM 2007 and 2014 Studies. In step 1, the linear relationship between PRS_BMI_ and anthropometric measures, in step 2, the relationship between PRS_BMI_ and PHDS, and in step 3, the relationship between PHDS and anthropometric measures was tested. Values are β-coefficients with standard errors and p-values. Model 1: adjusted for age, sex and baseline BMI in the association between PRS_BMI_ and percentual BMI change or between PHDS and percentual BMI change. Model 2: Model 1 further adjusted for education, smoking status and leisure-time physical activity.

Regarding the assumptions for mediation analyses, positive linear associations in step 1, between the PRS_BMI_ and baseline BMI (Model 2; β 1.42 (SE ± 0.08), *p* < 0.001) and BMI change (Model 2; β 0.36 (SE ± 0.15), *p* = 0.01), were found (Fig. 2). However, we did not observe a significant association in step 2, between the PRS_BMI_ and the PHDS (Model 2; β 0.02 (SE ± 0.02), *p* = 0.42), nor in step 3 between the PHDS and baseline BMI (Model 2; β −0.09 (SE ± 0.07), *p* = 0.20) or BMI change (Model 2; β 0.094 (SE ± 0.11), *p* = 0.73). As these assumptions of linear relationship between independent variable and mediator (Step 2) and mediator and outcome variables (Step 3) were not fulfilled, the mediation analyses were not further performed. Likewise, when we tested assumptions of linearity between PRS_BMI_ and other anthropometric variables (baseline WC and changes in weight, WC, and body fat %; step 1), as well as PHDS and these anthropometric variables (Step 3), findings tended to be similar (Supplementary Fig. 1).

The results remained similar also after the exclusion of energy under-reporters and participants reporting any possibly confounding health problems.

### Moderation analysis

We did not find any interaction between the PRS_BMI_ and the PHDS in any of the associations between the PRS_BMI_ and anthropometric measures (*p* > 0.05) (Table 2). This suggests that overall diet quality assessed with the PHDS did not moderate the associations between genetic susceptibility for obesity and anthropometric measures at baseline or changes in anthropometric measures during the follow-up. After conducting sensitivity analyses, the results remained similar.

**Table 2:** Association between polygenic risk score for BMI (PRS_BMI_) and baseline obesity and <5% or ≥ 5% anthropometric changes according to the Planetary Health Diet Score tertiles in DILGOM 2007 and 2014 Studies.

	PRS_BMI_								p_interact_^a^
	1		3			5			
		Ref.		OR	95% CI		OR	95% CI	
	<30, n / ≥ 30, n (%)		<30, *n* / ≥ 30, *n* (%)			<30, *n* / ≥ 30, *n* (%)			
**Baseline BMI obesity**^b^	544/46 (8)		489/105 (18)			353/203 (37)			0.18
PHDS, 1st tertile	239/18 (7)		191/48 (20)			136/84 (38)			
Model 1^c^		1		3.46	1.94, 6.17		8.78	5.03, 15.34	
Model 2^d^		1		3.26	1.80, 5.90		7.77	4.38, 13.78	
PHDS, 3rd tertile	149/10 (6)		168/29 (15)			109/57 (34)			
Model 1		1		2.70	1.27, 5.75		8.33	4.05, 17.14	
Model 2		1		2.51	1.16, 5.45		8.74	4.16, 18.36	
	<90 or 100 cm, *n* / ≥90 or 100 cm, *n* (%)		<90 or 100 cm, *n* / ≥90 or 100 cm, *n* (%)			<90 or 100 cm, *n* / ≥90 or 100 cm, *n* (%)			
**Baseline WC obesity**^e^	462/128 (22)		393/201 (34)			279/277 (50)			0.55
PHDS, 1st tertile	204/53 (21)		157/82 (34)			105/115 (52)			
Model 1		1		1.97	1.51, 2.56		3.92	3.01, 5.11	
Model 2		1		2.01	1.31, 3.09		4.22	2.74, 6.49	
PHDS, 3rd tertile	120/39 (25)		129/68 (35)			84/82 (49)			
Model 1		1		1.79	1.11, 2.89		3.47	2.13, 5.65	
Model 2		1		1.78	1.08, 2.94		3.58	2.14, 5.98	
	<5%, *n* / ≥ 5%, *n* (%)		<5%, *n* / ≥ 5%, *n* (%)			<5%, *n* / ≥ 5%, *n* (%)			
**Weight change**	435/155 (26)		422/172 (29)			407/149 (27)			0.63
PHDS, 1st tertile	196/61 (24)		176/63 (26)			164/56 (25)			
Model 1		1		1.30	0.85, 1.99		1.48	0.94, 2.34	
Model 2		1		1.24	0.80, 1.92		1.48	0.93, 2.36	
PHDS, 3rd tertile	116/43 (27)		131/66 (34)			118/48 (29)			
Model 1		1		1.34	0.82, 2.17		1.10	0.65, 1.87	
Model 2		1		1.46	0.89, 2.41		1.08	0.62, 1.87	
**BMI change**	452/138 (23)		439/155 (26)			423/133 (24)			0.39
PHDS, 1st tertile	204/53 (21)		184/55 (23)			173/47 (21)			
Model 1		1		1.27	0.81, 1.99		1.34	0.82, 2.16	
Model 2		1		1.22	0.77, 1.92		1.33	0.82, 2.18	
PHDS, 3rd tertile	123/36 (23)		138/59 (30)			121/45 (27)			
Model 1		1		1.46	0.89, 2.40		1.34	0.78, 2.30	
Model 2		1		1.61	0.97, 2.73		1.29	0.74, 2.27	
**WC change (*n*** = **1081)**	162/74 (31)		138/70 (34)			133/58 (30)			0.50
PHDS, 1st tertile	56/23 (29)		48/27 (36)			54/21(28)			
Model 1		1		1.43	0.72, 2.84		1.09	0.52, 2.30	
Model 2		1		1.37	0.68, 2.76		1.15	0.54, 2.46	
PHDS, 3rd tertile	54/20 (27)		50/26 (34)			38/22 (37)			
Model 1		1		1.40	0.69, 2.86		1.82	0.84, 3.95	
Model 2		1		1.46	0.71, 3.03		1.84	0.83, 4.10	
**Fat% change (*n*** = **1020)**	126/105 (46)		104/88 (46)			100/75 (43)			0.54
PHDS, 1st tertile	42/35 (46)		36/32 (47)			42/27 (39)			
Model 1		1		1.23	0.62, 2.44		1.15	0.57, 2.35	
Model 2		1		1.27	0.63, 2.56		1.23	0.59, 2.59	
PHDS, 3rd tertile	43/28 (39)		39/32 (45)			27/28 (51)			
Model 1		1		1.26	0.61, 2.61		2.06	0.93, 4.56	
Model 2		1		1.33	0.63, 2.81		1.98	0.87, 4.45	

*BMI* body mass index, *Fat%* body fat percentage, *PHDS* Planetary Health Diet Score, *PRS*_*BMI*_ Polygenic Risk Score for BMI, *WC* waist circumference.

^a^Interaction was tested by adding an interaction term (PRS_BMI_ divided into quintiles * PHDS divided into tertiles) into Model 2 of logistic regression testing the association between outcome variable and PRS_BMI_ (divided into quintiles) while adjusting the model with PHDS (divided into tertiles).

^b^Obesity defined as BMI ≥ 30 kg/m^2^.

^c^Model 1: adjusted for age, sex, and baseline anthropometric measure (weight/BMI/WC/Fat%) depending on which anthropometric change was examined. In addition, when testing weight changes, model was adjusted for baseline height.

^d^Model 2: Model 1 was further adjusted for education, smoking status, and leisure-time physical activity.

^e^Obesity defined as WC ≥ 90 cm for women and WC ≥ 100 cm for men.

### Results from replicated analyses

We replicated the moderation and mediation analyses in the Health 2000 and 2011 data samples (*n* = 2834, 54% of women, mean age at baseline 50 (SD ± 12) years) with four years longer follow-up than in DILGOM (Supplementary Table 1). In the replication analyses, the results on the PHDS’ mediating and moderating role in the associations between the PRS_BMI_ and anthropometric variables were similar to the original analyses (Supplementary Fig. 2, Supplementary Table 2).

## Discussion

This is the first study to explore how overall diet quality, based on the PHD and dietary intake assessed through an FFQ, mediates or modifies the relationship between genetic susceptibility to obesity and anthropometric measures. Our findings showed no evidence that diet quality according to the PHD influenced the associations between the PRS_BMI_ and anthropometric measures, either at baseline or during follow-up, in Finnish adults.

Previous studies using other diet quality indices have reported contradictory results regarding to mediating role of overall diet quality in the associations between genetic susceptibility to obesity and anthropometric measures [9, 15, 20]. Similar to our results, in a French-Canadian study (*n* = 7037 at baseline, and *n* = 2258 at follow-up; aged 55.6 ± 7.7 years) three different plant-based dietary indices (overall, healthy, or unhealthy) based on food group consumption collected with FFQ did not mediate the association between the PRS (based on 2 million SNP) and obesity outcomes cross-sectionally or longitudinally [15]. Likewise, in a Finnish cross-sectional twin study (*n* = 3977, mean age 34.1 years), did not find that the dietary score, reflecting diet quality based on Nordic and Finnish nutrition recommendations and assessed through a short qualitative FFQ mediate the association between the PRS for BMI (based on 1 million SNP) and anthropometric measures [9]. On the other hand, in another cross-sectional French-Canadian study (*n* = 750, aged 41.5 ± 14.9 years), poor diet quality, assessed with the Nutrient Rich Food (NRF) Index 6.3 and dietary data collected with three-day food records, appeared to partly mediate the association between the PRS_BMI_ (based on 523,101 SNP) and BMI and WC [20]. In our study, the assumption for mediation analysis was not met, as no linear association was found between the independent variable (PRS_BMI_) and the mediator (PHDS), aligning with previous research that also did not find a significant association between PRS and any plant-based dietary indices [15]. However, an inverse association was observed between PRS and the NRF Index 6.3, which is probably due the ability of this index to capture specific food quality aspects more effectively, as the authors noted [20]. This aligns with findings from studies that assessed the mediation effect of individual food groups, which concluded that certain food groups, such as meat, mediate the relationship between PRS and body fat percentage [15].

As with mediation studies, findings have been inconsistent regarding the modifying role of diet quality on the associations between genes and anthropometric measures. In some studies, especially participants with higher genetic risk for obesity have experienced less weight gain with a healthy diet [16, 18] while in other studies healthy diets have been associated with reduced weight gain independently of genetic susceptibility [15, 19, 21]. For example, in the Canadian study (*n* = 6087) adults, aged 55 ± 8 years was found an inverse association between the Healthy Eating Index adapted for the Canadian population (HEI-C, score range 0−100 points) and baseline anthropometric measures, particularly in participants with a higher PRS (including 97 BMI-associated SNP) [18]. Also, increasing adherence to the Alternate Healthy Eating Index 2010 (AHEI-2010) and Dietary Approach to Stop Hypertension (DASH) was associated with lower weight gain in two data sets of US female and male health professionals (women, *n* = 8828; men, *n* = 5218) with higher genetic susceptibility to obesity (based on 77 SNP) in a 20-year follow-up, however, no such association was found with better adherence to the Alternate Mediterranean Diet [16]. This was likely due to the narrow total score range of the index; in an AMED, the total score range was 0−9 points, whereas in the AHEI-2010 and DASH the range was 0−110 and 8−40 points, respectively [16]. In our datasets, score ranged from 1 to 11 (total score range was 0−13), and adherence to the diet was generally low (mean range of 3.8 points), which is possible due to the lack of awareness of the environmental impact of diets early 2000s when our data was collected as it is today. Narrow score range does not enable to distinguish participants from each other’s which could explain why we did not observe significant differences between groups based on their adherence to the PHDS or any associations in the mediation analyses between overall diet quality and anthropometric measures.

Furthermore, no significant gene-diet interaction was found when adherence to the AHEI (total score ranging from 2.5 to 77.5 points), two different Mediterranean diet scores (total score range 0−8 or 0−9 points) or the a posteriori dietary patterns were assessed in relation to obesity measures in cross-sectional and prospective analyses in middle-aged Swiss adults (*n* = 2542, aged 58.4 ± 10.6 years) [19]. The authors assumed that the relatively small sample size and high mean age of the participants could have influenced the results. Nevertheless, in a study of 11,735 British adults (aged 55.3 ± 7.6 years) no interaction was found, despite the large study population, between PRS including 97 obesity-related variants and dietary patterns in relation to obesity risk [21]. In our datasets, participants with a higher genetic risk for obesity were equally likely to gain weight during the follow-up as those who had the lowest genetic risk, even though they were more likely to live with obesity at baseline. This could be explained by the age of the participants as it is known that people carrying higher number of obesity-related variants gain weight earlier in life compared to those with lower genetic risk [51, 52]. For instance, a meta-analysis of 114 studies involving individuals of European descent found that most age-related BMI loci had stronger effects in younger participants ( ≤ 50 years) compared to older ones ( > 50 years) [51]. Furthermore, Masip et al. [15] did not find gene-diet interaction between genetic risk score including two million SNP and a priori plant-based dietary patterns (*n* = 7037, and *n* = 2258 at follow-up) similar to our results as the PHD emphasises plant-based foods.

The current study has several strengths and limitations. Regarding strengths, we used a validated FFQ and conducted different but comprehensive statistical analyses (mediation and moderation) both cross-sectionally and longitudinally with multiple anthropometric parameters as outcomes. However, we acknowledge the limitations associated with the FFQ including misreporting and memory bias. To consider the effect of misreporting on our results we conducted sensitivity analyses by excluding energy under-reporters from our analyses which did not affect the results [49, 50]. However, excluding energy under-reporters does not necessarily eliminate participants who may have overestimated their dietary intake, which could have influenced their overall diet quality and weakened the associations between diet quality and anthropometric changes. In addition, PRS_BMI_ was positively associated with baseline anthropometrics and anthropometric changes, indicating that it worked as we expected. However, we are familiar of the recent finding that current polygenic risk scores have a rather low predictive performance alone, and to improve their effectiveness, analyses will require other factors such as demographic, environmental, clinical, and molecular markers alongside the polygenic risk score in the future [6]. We also replicated all analyses in an independent population-based dataset with 11 years of follow-up. The results remained similar in the replication analyses, which provides consistency to our results. Lastly, our results are generalisable only to other populations of European descent as our polygenic score was based on the UK biobank genome-wide association study, which are based on European populations [6].

## Conclusion

In this study, we did not find evidence that overall diet quality based on the PHD either mediated or moderated the associations between the PRS_BMI_ and anthropometric measures at baseline or changes in anthropometric measures during the follow-up in the Finnish adult population. The relatively low adherence to the PHD and a relatively high mean age could have influenced the findings.

## Supplementary information

Supplemental material

## Data Availability

The datasets used in this research are available upon request through the Findata permit procedure at https://www.findata.fi/en/.
