# Supplementary material for: Role of Planetary Health Diet in the association between genetic susceptibility to obesity and anthropometric measures in adults
Source: Int J Obes (Lond). 2024 Oct 17;49(2):286–94. doi: 10.1038/s41366-024-01656-7 (PMC11805706; doi:10.1038/s41366-024-01656-7)
Supplement: Supplementary file 1 — Supplemental material [file 41366_2024_1656_MOESM1_ESM.pdf]

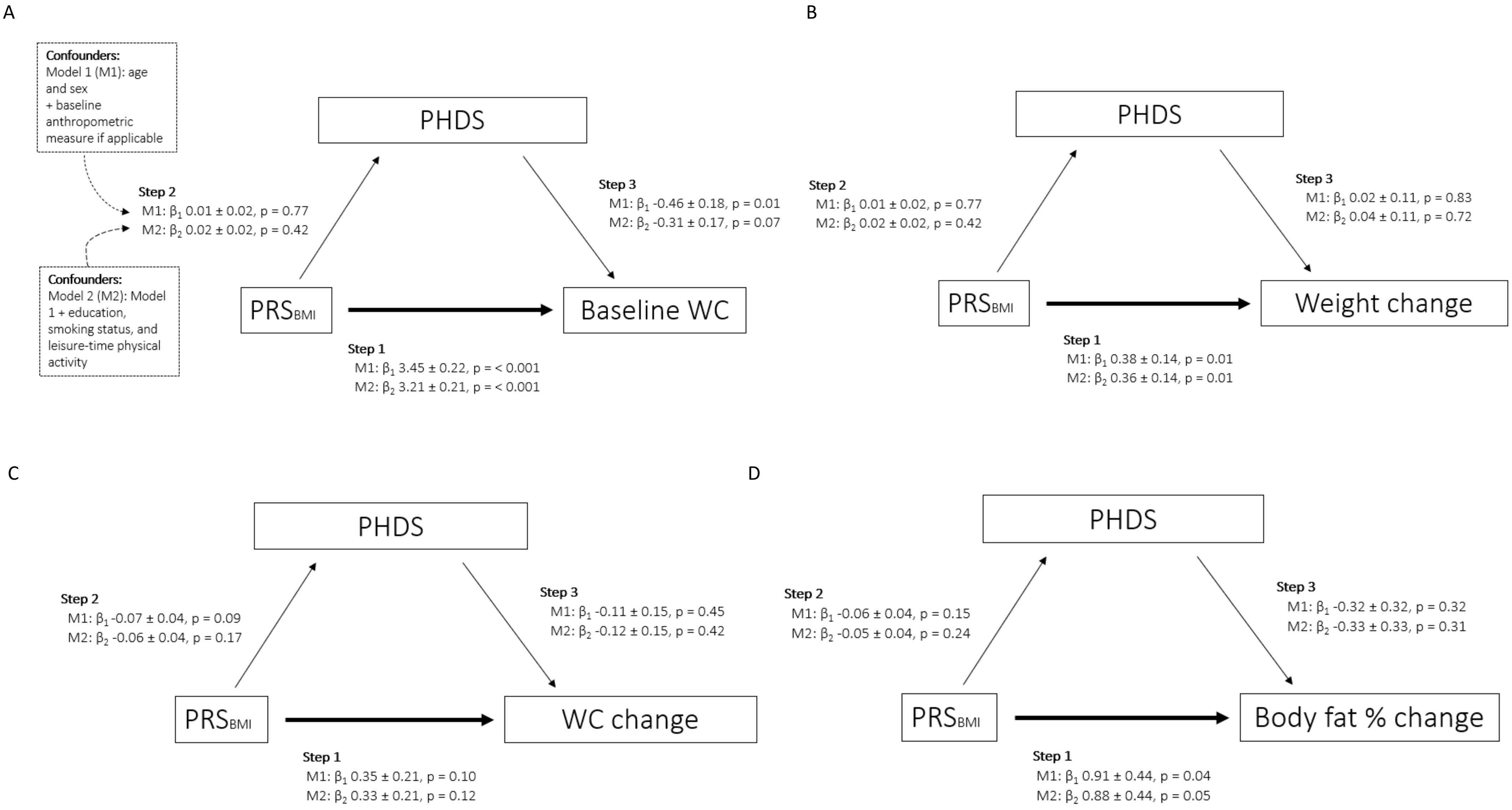

Supplemental figure 1. Unstandardised coefficients (β1, Model 1, and β2, Model 2) for linear regression analyses between main variables; polygenic risk score for body mass index, PRS_{BMI}, Planetary Health Diet Score (PHDS) and anthropometric measures; A) baseline waist circumference (WC), B) percentual change measured with weight, C) percentual change measured with WC, and D) percentual change measured with body fat percentage in DILGOM 2007 and 2014 Studies. In step 1 was tested the linear relationship between PRS_{BMI} and anthropometric measures, in step 2 the relationship between PRS_{BMI} and PHDS, and in step 3 the relationship between PHDS and anthropometric measures.

Values are β-coefficients with standard errors and p-values.

Model 1 is adjusted for age, sex, and baseline weight/WC/BF% depending on which of these was examined, and weight change additionally with baseline height.

Model 2 is Model 1 further adjusted for education, smoking status, and leisure-time physical activity.

Supplemental table 1. Baseline and follow-up characteristics of all participants and participants according to PRS for BMI (PRS_{BMI}) quintiles in Health 2000 and 2011 Studies.

		PRS_{BMI}				
	All	1	2	3	4	5
Number of participants (%)	2834	577 (20)	569 (20)	598 (21)	554 (20)	536 (19)
Women (%)	1535 (54)	315 (55)	292 (51)	337 (56)	305 (55)	286 (53)
**Baseline**						
Age, years	50 (12)	50 (12)	50 (12)	50 (11)	50 (12)	49 (11)
Low education level[1], %	32	27	27	33	34	38
Current smokers, %	24	18	27	22	22	29
Leisure-time physical inactivity[2], %	22	20	20	20	23	18
Energy intake, kJ	9548 (3150)	9582 (3001)	9761 (3196)	9536 (3145)	9272 (3168)	9587 (3234)
Energy intake, kcal	2282 (753)	2290 (717)	2333 (764)	2279 (752)	2216 (757)	2291 (773)
Planetary Health Diet Score[3]	3.5 (1.2)	3.6 (1.2)	3.4 (1.1)	3.6 (1.2)	3.6 (1.1)	3.5 (1.2)
1st tertile (0-3 points), %	51	50	53	49	50	53
2nd tertile, (4 points), %	30	29	32	30	29	29
3rd tertile, (5-10 points), %	19	21	15	21	20	18
Height, cm	169.3 (9.4)	169.6 (9.3)	170.2 (9.1)	169.2 (9.4)	168.8 (9.7)	168.8 (9.4)
Weight, kg	76.5 (15.2)	71.3 (13.2)	75.0 (13.8)	76.0 (14.6)	78.1 (15.4)	82.8 (16.7)
BMI, kg/m^2	26.6 (4.4)	24.7 (3.5)	25.8 (3.9)	26.5 (4.1)	27.3 (4.4)	29.0 (5.0)
Waist circumference, cm						
Men	97.4 (10.9)	93.9 (9.9)	95.2 (9.6)	96.9 (10.5)	99.1 (11.2)	102.2 (12.1)
Women	86.7 (12.7)	81.9 (9.9)	85.0 (12.0)	86.7 (12.4)	87.9 (12.9)	92.2 (13.9)
**Follow-up**						
Weight change, kg	1.2 (7.4)	0.7 (5.7)	1.3 (6.9)	1.4 (7.7)	0.8 (8.2)	1.7 (8.2)
n ≥ 5% (%)	885 (31)	163 (28)	174 (31)	204 (34)	164 (30)	180 (34)
BMI change, kg/m^2	0.6 (2.6)	0.4 (2.0)	0.6 (2.4)	0.7 (2.7)	0.5 (2.8)	0.8 (2.9)
n ≥ 5% (%)	962 (34)	179 (31)	184 (32)	217 (36)	180 (32)	202 (38)
Waist circumference change, cm[4]	2.2 (7.5)	1.7 (6.2)	2.3 (7.5)	2.5 (7.8)	1.8 (7.6)	2.9 (8.2)
n ≥ 5% (%)	830 (34)	145 (29)	165 (32)	191 (37)	162 (36)	167 (38)

kJ, kilojoules; kcal, kilocalorie; BMI, body mass index
Data are presented as means with standard deviations for continuous variables or percentages for categorical variables.
[1] Lower than upper secondary school or vocational school
[2] Leisure-time physical inactivity; light activities, like reading and watching television.
[3] Score could range from 0 to 13 points.
[4] n = 2430, PRS quintiles: n_1 = 497, n_2 = 511, n_3 = 521, n_4 = 456, n_5 = 445

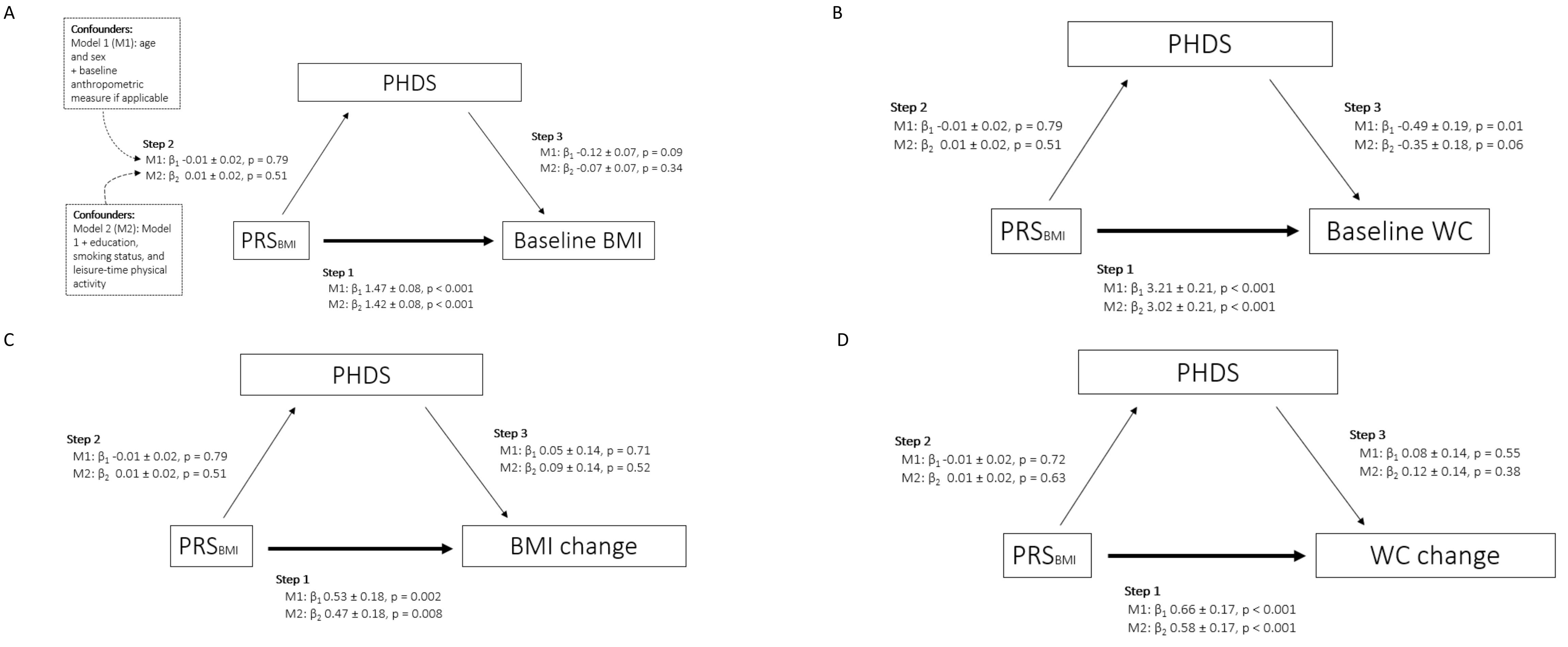

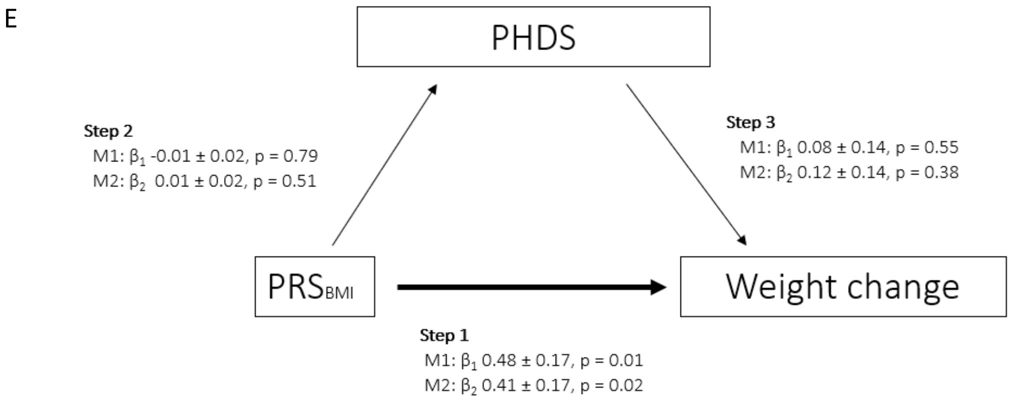

Supplemental figure 2. Unstandardised coefficients (β1, Model 1, and β2, Model 2) for linear regression analyses between main variables; polygenic risk score for body mass index, PRS_{BMI}, Planetary Health Diet Score (PHDS) and anthropometric measures; A) baseline body mass index (BMI), B) baseline waist circumference (WC), C) percentual change measured with BMI, D) percentual change measured with WC, and E) percentual change measured with weight in Health 2000 and 2011 Studies. In step 1 was tested the linear relationship between PRS_{BMI} and anthropometric measures, in step 2 the relationship between PRS_{BMI} and PHDS, and in step 3 the relationship between PHDS and anthropometric measures.

Values are β-coefficients with standard errors and p-values.

Model 1 is adjusted for age, sex, and baseline weight/WC/BF% depending on which of these was examined, and weight change additionally with baseline height.
Model 2 is Model 1 further adjusted for education, smoking status, and leisure-time physical activity.

Supplemental table 2. Association between polygenic risk score for BMI (PRS_{BMI}) and baseline obesity and <5% or ≥ 5% anthropometric changes according to the Planetary Health Diet Score tertiles in Health 2000 and 2011 Studies.

	PRS_{BMI}								
	1		3			5			
		Ref.		OR	95% CI		OR	95% CI	$p_{interact}$
	<30, n / ≥ 30, n (%)		<30, n / ≥ 30, n (%)			<30, n / ≥ 30, n (%)			
**Baseline BMI obesity[1]**	535/42 (7)		489/109 (18)			332/204 (38)			0.96
PHDS, 1st tertile	269/22 (8)		236/58 (20)			176/109 (38)			
Model 1		1		2.95	1.74, 5.01		8.35	5.04, 13.84	
Model 2		1		1.94	1.77, 5.13		8.40	5.04, 13.99	
PHDS, 3rd tertile	113/8 (7)		105/21 (17)			61/36 (37)			
Model 1		1		2.96	1.25, 7.04		8.95	3.86, 20.72	
Model 2		1		3.23	1.34, 7.75		9.29	3.94, 21.89	
	<90 or 100cm, n / ≥ 90 or 100 cm, n (%)		<90 or 100cm, n / ≥ 90 or 100 cm, n (%)			<90 or 100cm, n / ≥ 90 or 100 cm, n (%)			
**Baseline WC obesity[2]**	456/121 (21)		390/208 (35)			261/275 (51)			0.24
PHDS, 1st tertile	230/61 (21)		181/113(38)			151/134 (4)			
Model 1		1		2.36	1.61, 3.45		3.80	2.59, 2.57	
Model 2		1		2.42	1.64, 3.56		3.75	2.54, 5.52	
PHDS, 3rd tertile	99/22 (18)		86/40 (32)			47/50 (52)			
Model 1		1		2.22	1.21, 4.09		5.22	2.79, 9.80	
Model 2		1		2.26	1.22, 4.21		5.11	2.69, 9.73	
	<5 %, n / ≥ 5 %, n (%)		<5 %, n / ≥ 5 %, n (%)			<5 %, n / ≥5 %, n (%)			
**Weight change**	414/163 (28)		394/204 (34)			356/180 (34)			0.59
PHDS, 1st tertile	214/77 (27)		196/98 (33)			192/93 (33)			
Model 1		1		1.76	1.19, 2.56		1.66	1.11, 2.48	
Model 2		1		1.74	1.18, 2.56		1.55	1.03, 2.32	
PHDS, 3rd tertile	89/32 (26)		78/48 (38)			60/37 (38)			
Model 1		1		1.78	1.00, 3.17		1.88	0.99, 3.59	
Model 2		1		1.91	1.06, 3.44		2.01	1.04, 3.88	
**BMI change**	3987145 (29)		381/217 (36)			334/202 (38)			0.41
PHDS, 1st tertile	207/84 (29)		194/100 (34)			175/110 (39)			
Model 1		1		1.54	1.06, 2.23		1.91	1.30, 2.80	
Model 2		1		1.50	1.03, 2.18		1.78	1.21, 2.63	
PHDS, 3rd tertile	86/35 (29)		71/55 (44)			60/37 (38)			
Model 1		1		1.93	1.11, 3.34		1.53	0.82, 2.86	
Model 2		1		1.99	1.14, 3.49		1.60	0.85, 3.01	
**WC change (n = 1081)**	352/145 (29)		330/191 (37)			278/167 (38)			0.74
PHDS, 1st tertile	182/72 (28)		161/94 (37)			147/95 (39)			
Model 1		1		1.76	1.20, 2.59		2.05	1.38, 3.05	
Model 2		1		1.78	1.21, 2.64		1.90	1.26, 2.84	
PHDS, 3rd tertile	76/29 (28)		69/42 (38)			45/31 (41)			
Model 1		1		1.65	0.91, 2.99		2.30	1.17, 4.50	
Model 2		1		1.73	0.94, 3.16		2.38	1.19, 4.75	

BMI, body mass index; PHDS, Planetary Health Diet Score; PRS_{BMI}, Polygenic Risk Score for BMI; WC, waist circumference

[1] Obesity defined as BMI ≥ 30 kg/m^2

[2] Obesity defined as WC ≥ 90 cm for women and WC ≥ 100 cm for men.

^ Interaction was tested by adding an interaction term (PRS divided into quintiles * PHDS divided into tertiles) into Model 2 of logistic regression testing the association between outcome variable and PRS_{BMI} (divided into quintiles) while adjusting the model with PHDS (divided into tertiles).

Model 1: adjusted for age, sex, and baseline anthropometric measure (weight/BMI/WC/Fat%) depending on which anthropometric change was examined. In addition, when testing weight changes model was adjusted for baseline height.

Model 2: Model 1 was further adjusted for education, smoking status, and leisure-time physical activity.
